# An oral quinoline derivative, MPT0B392, causes leukemic cells mitotic arrest and overcomes drug resistant cancer cells

**DOI:** 10.18632/oncotarget.15115

**Published:** 2017-02-06

**Authors:** Min-Wu Chao, Han-Li Huang, Wei-Chun HuangFu, Kai-Cheng Hsu, Yi-Min Liu, Yi-Wen Wu, Chao-Feng Lin, Yi-Lin Chen, Mei-Jung Lai, Hsueh-Yun Lee, Jing-Ping Liou, Che-Ming Teng, Chia-Ron Yang

**Affiliations:** ^1^ The Program for Cancer Biology and Drug Discovery, College of Medical Science and Technology, Taipei Medical University, Taipei 11031, Taiwan; ^2^ School of Pharmacy, College of Pharmacy, Taipei Medical University, Taipei 11031, Taiwan; ^3^ School of Pharmacy, College of Medicine, National Taiwan University, Taipei 10051, Taiwan; ^4^ Translational Research Center, Taipei Medical University, Taipei 11031, Taiwan; ^5^ Pharmacological Institute, College of Medicine, National Taiwan University, Taipei 10051, Taiwan

**Keywords:** MPT0B392, mitotic arrest, drug resistance, acute leukemia

## Abstract

Despite great advances in the treatment of acute leukemia, a renaissance of current chemotherapy needs to be improved. The present study elucidates the underlying mechanism of a new synthetic quinoline derivative, MPT0B392 (B392) against acute leukemia and its potential anticancer effect in drug resistant cells. B392 caused mitotic arrest and ultimately led to apoptosis. It was further demonstrated to be a novel microtubule-depolymerizing agent. The effects of oral administration of B392 showed relative potent anti-leukemia activity in an *in vivo* xenograft model. Further investigation revealed that B392 triggered induction of the mitotic arrest, followed by mitochondrial membrane potential loss and caspases cleavage by activation of c-Jun N-terminal kinase (JNK). In addition, B392 enhanced the cytotoxicity of sirolimus in sirolimus-resistant acute leukemic cells through inhibition of Akt/mTOR pathway and Mcl-1 protein expression, and also was active in the p-glycoprotein (p-gp)-overexpressing National Cancer Institute/Adriamycin-Resistant cells with little susceptibility to p-gp. Taken together, B392 has potential as an oral mitotic drug and adjunct treatment for drug resistant cancer cells.

## INTRODUCTION

Current therapy for most types of acute leukemia consists of traditional chemotherapy, including microtubule-targeting agents (MTA) such as vincristine [1–4]. MTA have been used for decades in a majority of malignancies, including prostate cancer, ovarian cancer, breast cancer, non-small lung cancer, and hematological malignances [5], and they continue to produce impressive clinical outcomes. Despite their reputation as old-fashioned drugs, many MTA have been developed and tested in ongoing clinical trials [6].

Resistance to chemotherapy has been an essential issue in current cancer treatment over the past cascades. The mechanisms of chemotherapeutic resistance, which can be divided into intrinsic and acquired, vary in different types or stages of cancers. Tumor cell-specific over-activated pathways and p-glycoprotein (p-gp) overexpression are the most common drug-resistance mechanisms in chemotherapy [7]. P-glycoprotein, a family member of ATP-binding cassette (ABC) transporters, pumps out its substrate, such as vincristine or paclitaxel, to decrease the concentrations of drugs within the cells, and which plays an essential role in MTA-caused drug resistance [8].

The mammalian target of rapamycin (mTOR), a key serine/theonine kinase, regulates various cellular processes required for protein synthesis, growth, and cell cycle progression and metabolism [9]. Genetic mutations, over-activated tyrosine kinase receptors, and amplification of key factors in PI3K/AKT pathways have been identified to lead to abnormal activation of mTOR signaling in leukemia [10]. Thus, mTOR inhibitors were investigated in preclinical and clinical antitumor studies, such as leukemia, lymphoma, endometrial carcinoma, renal carcinoma, breast cancer and glioblastoma [11].

Quinoline derivatives possess many diverse biological activities, including antifungal [12], antimalarial [13, 14], and anticancer effects [15–17]. The present study investigates the anticancer effect of a new synthetic quinoline compound, MPT0B392 (B392; 6-methoxy-2-(3,4,5-trimethoxy-benzenesulfonyl)-quinolin-5-ylamine), on acute leukemia and drug resistance. We found that B392 inhibited tubulin polymerization and induced c-Jun N-terminal kinase (JNK) activation, leading to apoptosis. More importantly, B392 can be administered orally in an *in vivo* leukemic cell xenograft model. Additionally, B392 potentiated cytotoxicity in sirolimus (rapamycin)-resistant acute leukemic cells and the multidrug resistant cell line. It was further be demonstrated not a p-gp substrate. The results of the present study suggest that B392, as a developmental drug, could have a potential use in the clinics, especially applied in second line chemotherapy.

## RESULTS

### Evaluating the anticancer effect of B392 in leukemic cell lines

**Figure 1:**
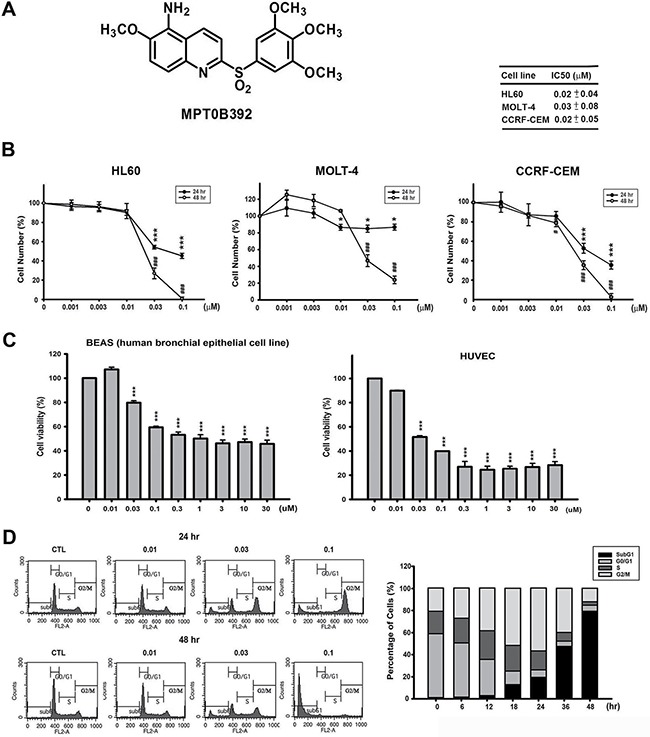
Chemical structure of B392 and its potential anticancer effect *in vitro* (**A**) The chemical structure of B392. (**B**) Cell viabilities of B392 in HL60 (acute promyelocytic leukemia), MOLT-4 (acute lymphoblastic leukemia), CCRF-CEM (acute lymphoblastic leukemia) and (**C**) normal cells (BEAS, human bronchial epithelial and HUVEC, human umbilical vein endothelial cell) were determined by MTT assay at 48 h. *P < 0.05, ***P < 0.001, ^#^*P* < 0.05, ^###^*P* < 0.001 compared with the control group. (**D**) Time (right panel) and concentration-dependent (left panel) of B392 on cell cycle progression. HL60 cells were treated with vesicle (0.1% DMSO) or 0.01, 0.03, 0.1 μM of B392 for 24 and 48 h (left panel) and treated with vesicle (0.1% DMSO) or 0.1 μM of B392 for 6, 12, 18, 24, 36, 48 h (right panel). Cell cycle distribution was performed by flow cytometry. IC50 values were calculated by sigmoidal dose-response equation and expressed as mean plus minus SD at 48 h treatment.

To determine the *in vitro* antitumor activity of B392 (Figure 1A), we performed an MTT assay in leukemic cell lines. As shown in Figure 1B, B392 inhibited the cell viability of HL60, MOLT-4, and CCRF-CEM cells in a concentration-dependent manner, with IC_50_ values of 0.02, 0.03, and 0.02 μM, respectively, at 48 h; and it shows better cytotoxic effect than vincristine in primary AML cells (Supplementary Figure 1A). Moreover, B392 caused less sensitivity in normal cells, including BEAS (human bronchial epithelial cells), HUVECs (human umbilical vein endothelial cells) (Figure 1C) and PBMC (peripheral blood mononuclear cell) (Supplementary Figure 1B). To investigate the mechanism underlying the cytotoxicity exhibited by B392, we further evaluated cell cycle progression after treatment with B392. The data show that B392 triggered cells arrest in the G2/M phase, followed by accumulation in subG1 phase in a concentration and time-dependent manner (Figure 1D; Supplementary Figure 2A–2C). In conclusion, B392 displayed more sensitive on leukemic cells than normal cells.

### B392 induces apoptosis in xenograft models

**Figure 2:**
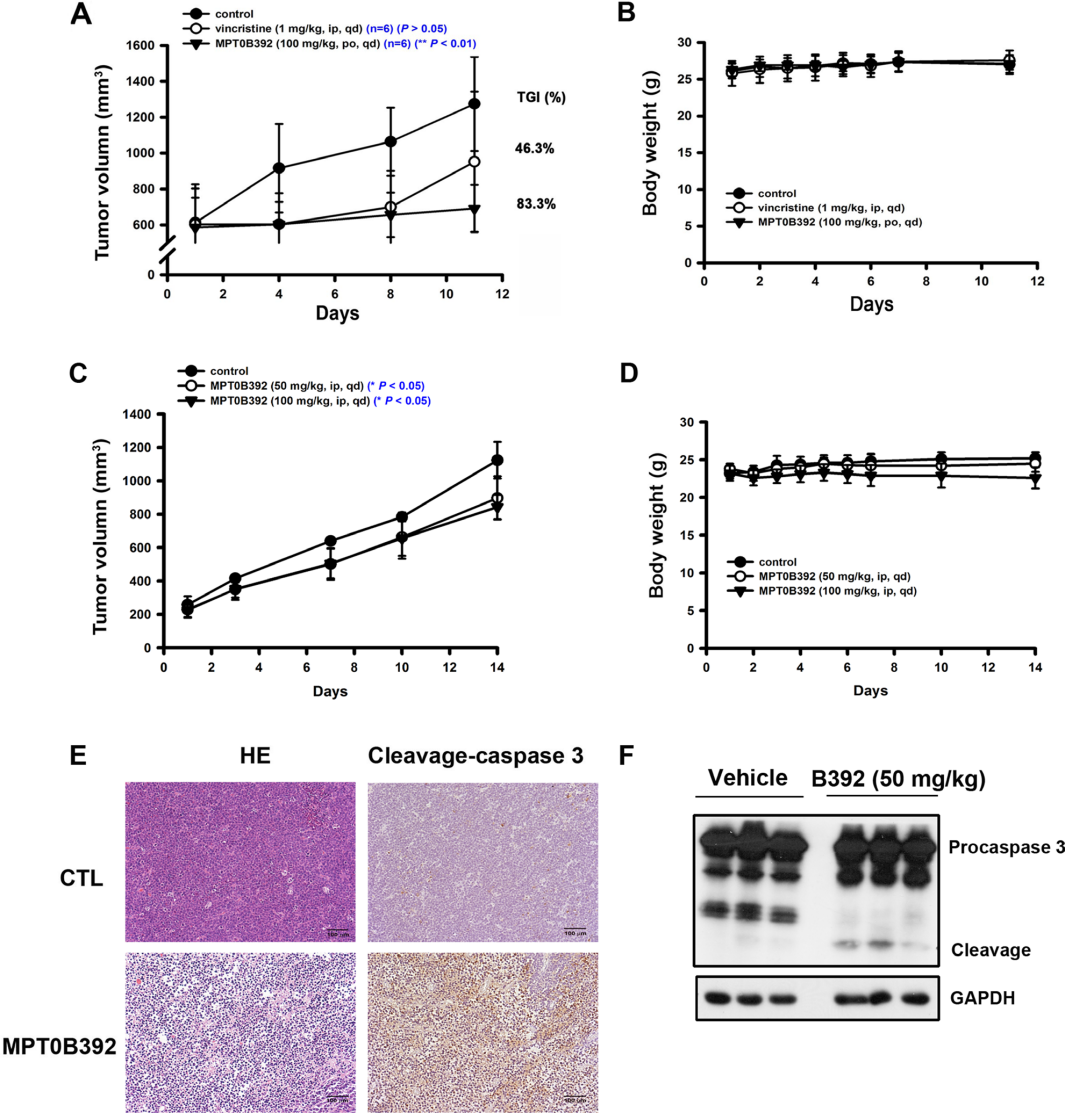
The effect of B392 in MOLT-4 and HL60 xenograft models SCID mice were ectopically implanted with MOLT-4 cells (**A**, **B**) or HL60 cells (**C**, **D**). Vincristine 1 mg/kg (i.p.), B392 50 mg/kg or 100 mg/kg (p.o. or i.p.) were treated. (A and C) The curves show the effect of B392 on tumor volume and percentage of tumor growth delay (TGD), which was calculated for treatment groups relative to control group. (B and D) The body weight of mice after indicated drugs treatment. (**E**) Immunohistochemical staining for the MOLT-4 tumors’ sections. Upper panel is the control, and lower panel is the B392 treatment group. Left panel was stained with hematoxylin and eosin; and right panel with cleavage caspase 3, which represents cells under apoptosis. Each tumor sample is under 160 ×magnification. (**F**) HL60 xenograft tumour homogenates were used to analyse cleavage caspase 3 protein expressions to determine apoptosis.

The MOLT-4 and HL60 xenograft model studies were performed to evaluate B392 as a potential anticancer drug for the treatment of leukemia. Figure 2A shows that B392 treatment resulted in significant tumor growth delay (83.3%) and tumor volume inhibition (*P* < 0.01) (Figure 2A, 2C), without loss of body weight (Figure 2B, 2D). Here, vincristine treatment group was used to be a positive control to demonstrate the animal studiesy were workable. Moreover, immunochemistry staining and tumor homogenates show that B392 treatment induced apoptosis in cancer cells, demonstrated by detection of positive staining of cleavage caspase 3 (Figure 2E, 2F). Taken together, these data suggest that B392 exhibited anticancer activity with less cytotoxicity both *in vitro* and *in vivo*.

### The effect of B392 on microtubule dynamics

**Figure 3:**
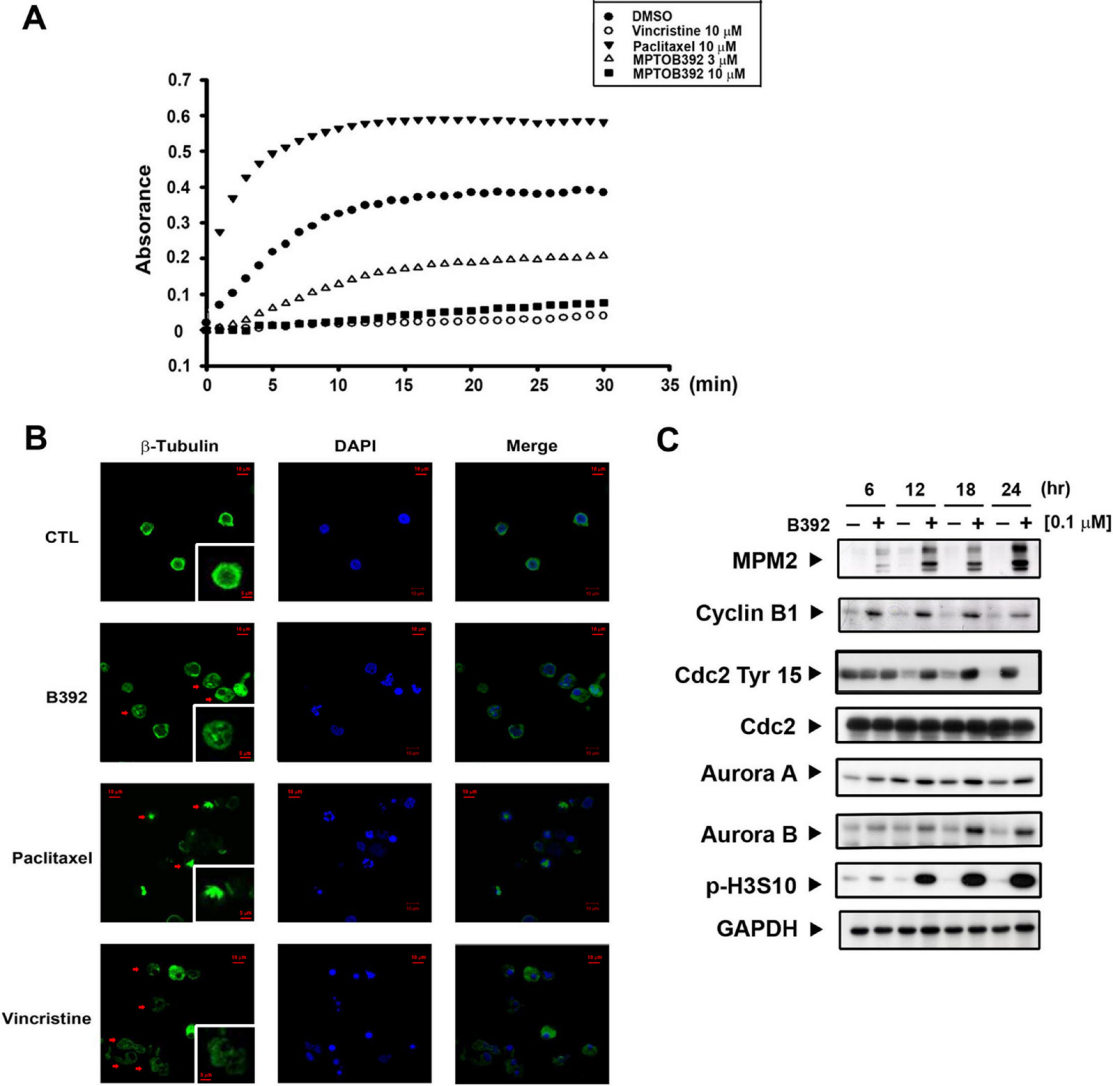
B392 is a depolymerizing agent and caused mitotic arrest (**A**) *In vitro* tubulin polymerization assay was performed to evaluate the effect of B392 on microtubule dynamics. In cell-free condition, tubulin proteins were in reaction buffer in the presence or absence of B392 (3 or 10 μM), paclitaxel (10 μM) or vincristine (10 μM). Assembly of microtubules was determined by measuring absorbance at 340 nm. (**B**) HL60 cells were exposed to 0.1 μM B392, 10 μM vincristine and 10 μM paclitaxel for 24 h. The changes of microtubule network were visualized by staining β-tubulin as arrows indicated. Nuclear DNA was stained by DAPI. 10 μM vincristine and 10 μM paclitaxel were used as positive controls for tubulin depolymerization and tubulin polymerization, respectively. (**C**) HL60 cells were treated 0.1 μM B392 time-dependently to detect the expressions of G2/M related proteins.

Microtubules play an important role in cell mitosis, and administration of microtubule-binding agents usually result in mitotic arrest [5]. Therefore, we used an *in vitro* tubulin-polymerization assay as well as tubulin staining (observed using confocal microscopy) to assess whether B392 had an effect on tubulin and would lead to cells accumulation in G2/M phase. As seen in Figure 3A, B392 caused tubulin depolymerization *in vitro* and disrupted microtubule formation, as observed by diffusion of stained tubulin into the cytoplasm, which phenomena was also shown in vincristine treatment. Paclitaxel and vincristine were utilized as positive controls for tubulin polymerization and depolymerization, respectively (Figure 3B).

To clarify the mechanism by which B392 induced G2/M cell cycle arrest in leukemic cells, we observed the expression of G2/M regulatory proteins. As cells enter mitosis, a variety of proteins are phosphorylated either directly or indirectly by M-phase-promoting factor (MPF) [18]. MPM2, an antibody that can specifically detect the phosphorylation of M phase regulatory proteins, is often used to differentiate the phase in which cells accumulate (e.g. G2 or M phase). The results show that B392 treatment upregulated intracellular MPM2 and cyclin B1 and downregulated the inhibitory Try-15 residue of Cdk1 (cdc2), indicating that B392 induced cell arrest in M phase. Additionally, increased expression of Aurora A and B (mitotic spindle kinases), which play a role in the regulation of cell division [19] as well as phosphorylation of H3S10, suggested that B392 might activate the mitotic checkpoint (Figure 3C; Supplementary Figure 2D). Our data suggests that B392 is a novel microtubule-destabilizing agent that induces mitotic arrest in leukemic cells.

### Evidence of B392-triggered apoptosis

**Figure 4:**
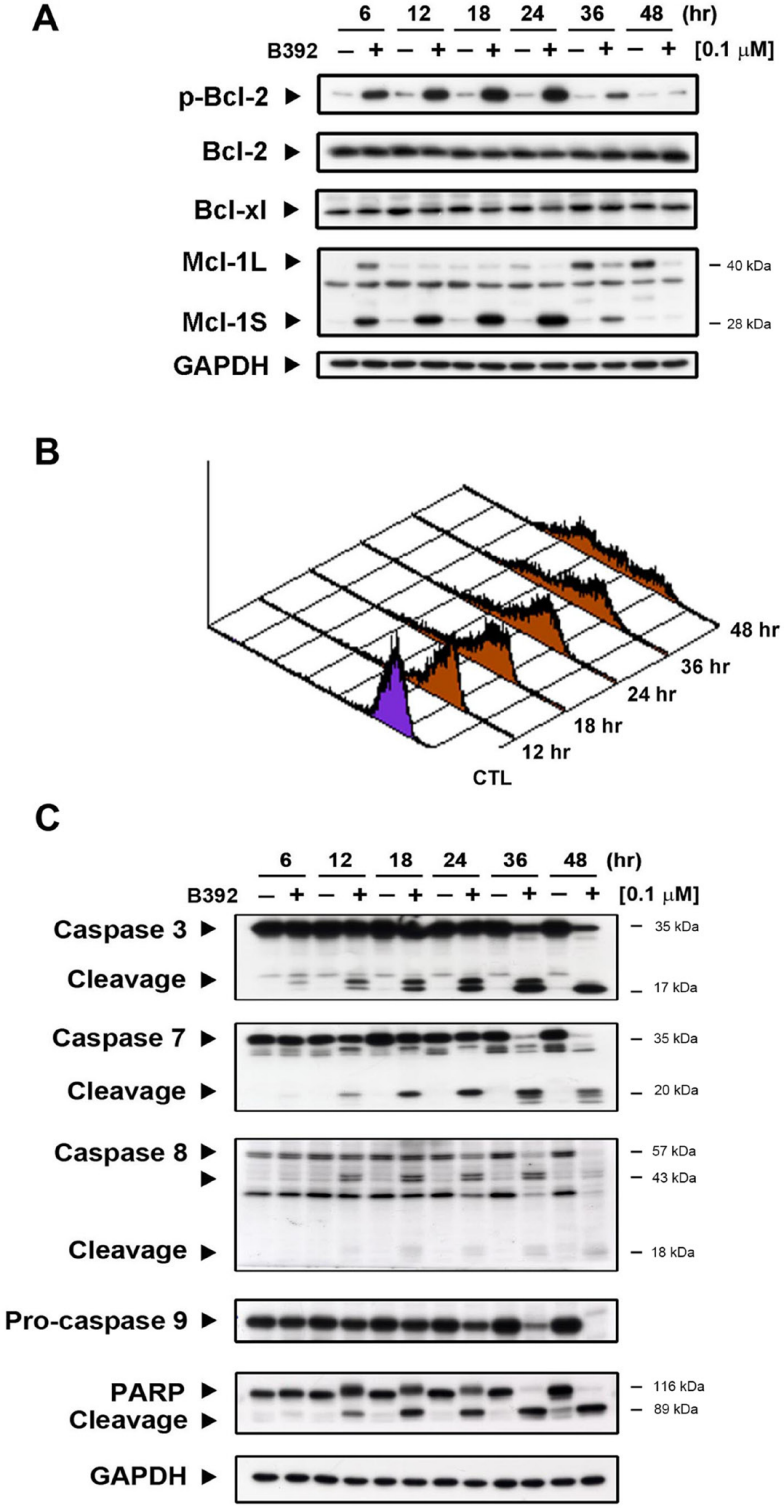
The effect of B392 on apoptosis (**A**) HL60 cells were treated with 0.1 μM B392 for indicated time then were harvested for detection of p-Bcl-2, Bcl-xl, Bcl-2, and Mcl-1 by western blot analysis. (**B**) The phenomena of mitochondria potential loss was measured by flow cytometry analysis with rhodamine-123. HL60 cells were treated with B392 (0.1 μM) for indicated time and then incubated with rhodamine-123 (10 μM) at 37°C for 30 min. The horizontal axis shows the relative fluorescence intensity, when the right curve shift to the left cure represents a loss of mitochondrial membrane potential. (**C**) HL60 cells were treated with vehicle (0.1% DMSO) or B392 (0.1 μM) for indicated times. The expressions of cleavage caspases and PARP were detected by western blot analysis. GAPDH used as an internal control.

Mitochondria play a crucial role in cells undergoing the apoptotic process [20]; hence, we further evaluated the expressions of mitochondrial proteins and mitochondrial function, which was assessed by measuring the permeability of their outer membranes. As seen in Figure 4A, B392 caused the phosphorylation of Bcl-2, Mcl-1S (Mcl-1 short form) increase and decrease in Mcl-1L (Mcl-1 long form), which provided evidence that mitochondrial membrane potential time-dependently lost in HL60 cells (Figure 4B). Mcl-1S, a splicing variant of the antiapoptotic Mcl-1 (Mcl-1L), is a proapoptotic protein [21]. Induction of apoptosis by B392 was elucidated by the observation of a hypodiploid peak (subG1) in leukemic cells (Figure 1D). The activation of caspase 3, 7, 8, 9 and poly (ADP-ribose) polymerase (PARP) cleavage in a time-dependent manner further confirmed B392-induced apoptosis (Figure 4C). These data suggest that B392 has an effect on mitochondrial proteins, membrane potential as well as activation of the classic apoptosis pathway and ultimately cell death.

### JNK activation plays a key role in B392-induced apoptosis of leukemic cells

**Figure 5:**
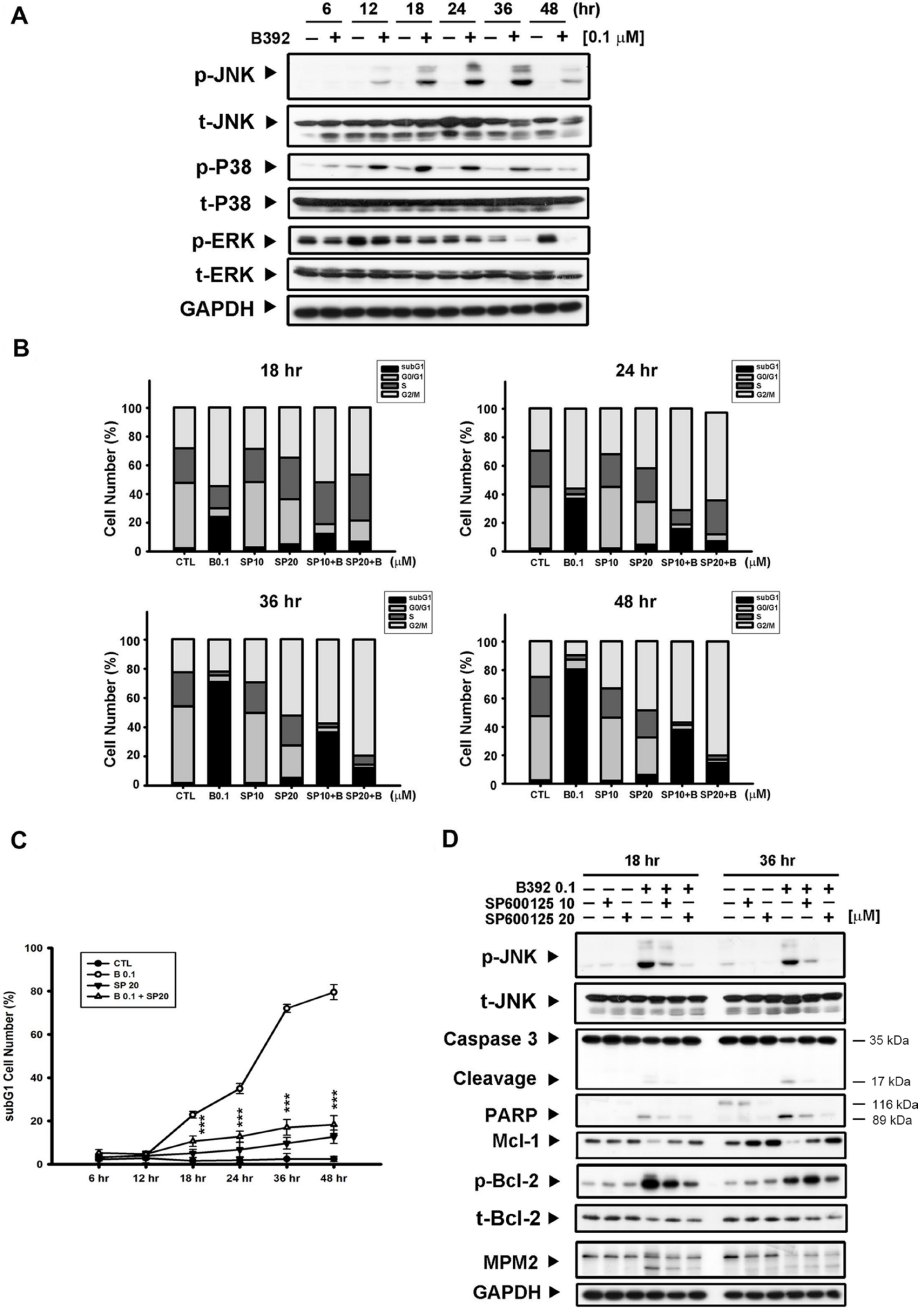
JNK activation was involved in B392-caused cell apoptosis (**A**) HL60 cells were treated with vehicle (0.1% DMSO) or B392 (0.1 μM) for time course. The cells were harvested for detection of indicated proteins by western blot analysis. (**B**) HL60 cells were exposed to B392 (0.1 μM) in the absence or presence of SP600125 (10, 20 μM) for 18, 24, 36 and 48 hours. Cell cycle distribution was analyzed by flow cytometry as well as determining (**C**) the percentage of subG1 cell number. (***P < 0.001) (**D**) The expression of indicated proteins was determined after treatment of HL60 with 0.1 μM B392 in the absence or presence of SP600125 (10, 20 μM) pretreatment for 30 min. SP: SP600126, p-JNK inhibitor.

It has been reported that differential regulation of mitogen-activated protein kinases (MAPKs) is induced by microtubule binding agents [22, 23]. To further investigate the mechanism of B392-induced cell apoptosis, we analyzed the phosphorylation of JNK, P38, and extracellular signal-regulated kinase (ERK) after treatment with B392. The results show that p-JNK and p-P38 were induced by B392 in a time-dependent manner, with maximum induction observed at 24 and 36 h (Figure 5A). We used a p-P38 inhibitor (SB203580) and p-JNK inhibitor (SP600125) to identify the specific MAPK involved in B392-induced cell death. The data on cell cycle distribution revealed that only SP600125 was able to reverse B392-induced subG1 accumulation (Figure 5B, 5C; Supplementary Figure 3) as well as the expressions of mitochondrial proteins (p-Bcl-2, and Mcl-1), caspase 3, and PARP (Figure 5D) in HL60 cells. The above observation was not found after co-treatment with SB203580 and B392 (Data not shown).

### B392 enhanced the cytotoxicity of sirolimus in a sirolimus-resistant cell line

**Figure 6:**
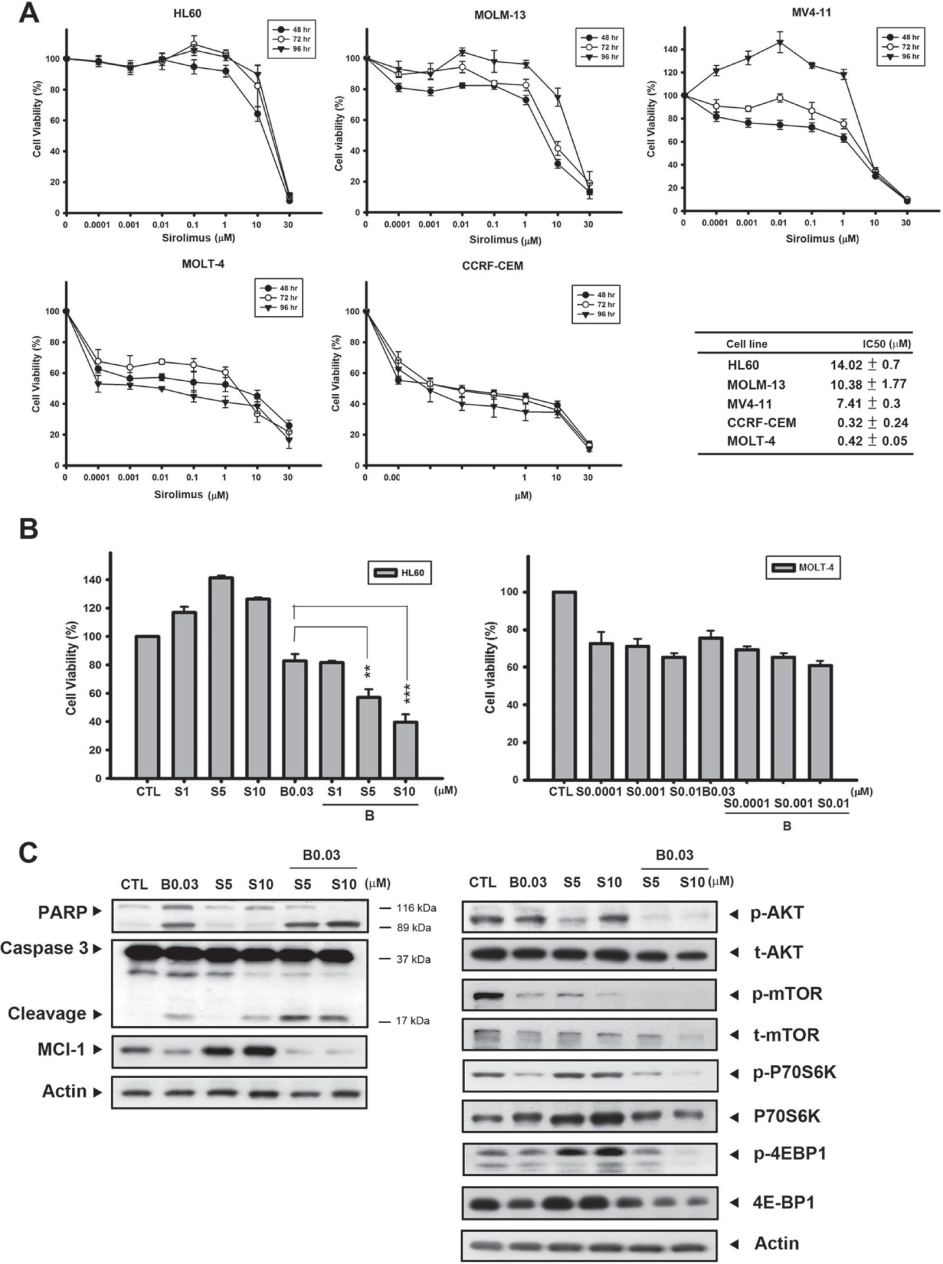
B392 sensitized sirolimus anticancer activity in sirolimus-resistant cell line (**A**) Acute myeloid leukemia cell lines (HL60, MV4-11, MOLM-13) and lymphoblastic cell lines (MOLT-4, CCRF-CEM) were treated with indicated concentration of sirolimus for 48, 72, 96 h. The cell viability was determined by MTT assay. The right table summarizes the IC50 of sirolimus at 72 h. IC50 values were calculated by sigmoidal dose-response equation and expressed as mean plus minus SD. (**B**) The cell viability of B392 combined with sirolimus at 72 h. (**C**) The expression of caspase 3, PARP, p-AKT, t-AKT, p-mTOR, t-mTOR, p-P70S6K, P70S6K, p-4EBP1, 4EBP, Mcl-1 and actin was determined after 0.03 μM B392 combination with 5 μM or 10 μM sirolimus for 72 h. S: sirolimus **P < 0.01, ***P < 0.001.

It has been shown that a number of genetic mutations elevate PI3K/AKT/mTOR signaling, and contribute to cell proliferation, survival, and drug resistance in leukemia [24, 25]. Thus, combining cytotoxic chemotherapy agents could enhance sensitivity to mTOR inhibitors and achieve better outcomes. We first evaluated the baseline sensitivity of sirolimus (rapamycin) in different acute leukemic cells lines via the MTT assay. Figure 6A shows that acute myeloid leukemic cells HL60, MOLM-13, and MV4-11 were more resistant to sirolimus, with IC_50_ values of 14.02, 10.38, and 7.41 μM, respectively, while comparing with acute lymphoblastic leukemic cells MOLT-4 and CCRF-ECM (IC_50_ values of 0.32 and 0.42 μM, respectively). In the sirolimus-resistant cell line, HL60, a combination of sirolimus with B392 enhanced the cytotoxicity of sirolimus compared to sirolimus alone; however, this phenomenon was not observed in the sirolimus-sensitive cell line, MOLT-4 (Figure 6B; Supplementary Table 1). The combination of B392 and sirolimus clearly induced PARP and caspase 3 cleavage, as well as downregulated a key prosurvival protein, Mcl-1 (Figure 6C, *left panel*). The effect of a sirolimus-B392 combination on AKT/mTOR signaling was also determined. B392 alone only had a slight effect on the AKT/mTOR pathway; however, the combination dramatically decreased the expression of p-AKT and p-mTOR, as well as mTOR's downstream targets, p-P70S6K and p-4EBP1 (Figure 6C, *right panel*). Taken together, B392 can strengthen the effect of sirolimus on mTOR signaling and can enhance cytotoxicity in sirolimus-resistant acute myeloid leukemia (AML) cells.

### Multiple drug resistance cell line was sensitive to B392, which was not a p-gp substrate

**Figure 7:**
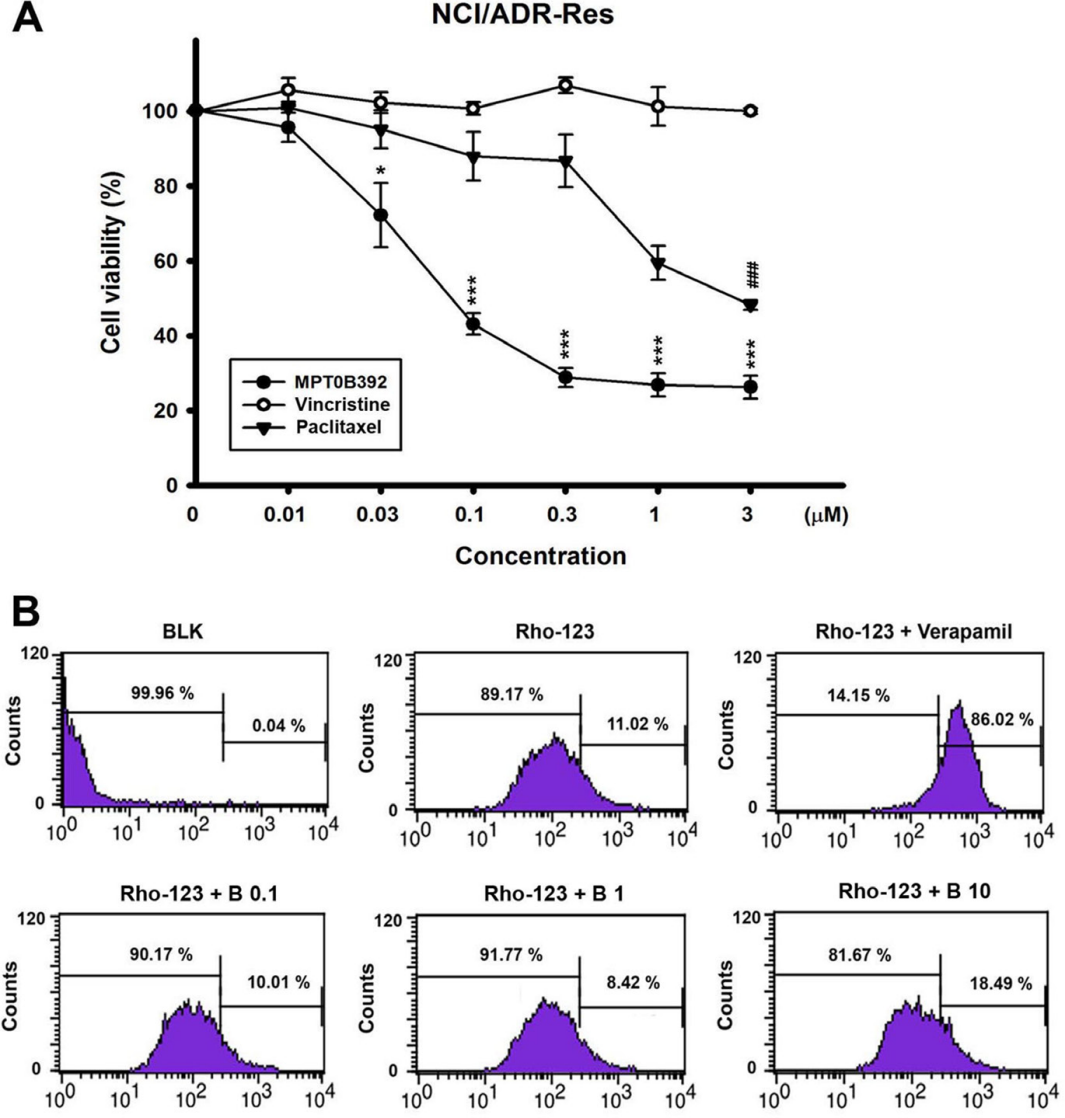
The cell viability of B392 on NCI/ADR-RES cell line and the effect on p-gp activity (**A**) NCI/ADR-RES cells were treated with indicated concentrations of B392, vincristine or paclitaxel for 48 h. The cell viability was determined by MTT assay. (*P < 0.05, **P < 0.01, ***P < 0.001) (**B**) NCI/ADR-RES cells were treated with or without indicated agents (Verapamil, 50 μM; B392, 0.1, 1, 10 μM) for 1 h. After treatment with indicated drugs for 30 min, 10 μM Rhodiamine-123 was added and incubation for 30 min at 37°C. The signal of fluorescence was detected by flow cytometry. BLK, blank; Rho-123, Rhodiamine 123; B, B392.

The p-gp multidrug transporter, a broad ATP-dependent efflux pump, can export a variety of natural products and microtubule-binding agents, including vincristine and paclitaxel, from the cell; it reduces the efficacy of anticancer drugs and is often regarded as a major mechanism for drug resistance. Figure 7A shows that p-gp-overexpressed NCI/ADR-RES cell was more sensitive to B392 than to vincristine or paclitaxel. Additionally, we investigated the susceptibility of B392 to p-gp in NCI/ADR-RES cell line. The results indicate that rhodamine-123, a substrate of p-gp, was effluxed, and this phenomenon was inhibited by co-treatment with verapamil, a p-gp inhibitor, as observed by the dye retention in the cells. In contrast, the efflux of rhodamine did not decrease even after co-incubation with a high concentration of B392 (10 μM), implying that the intake of B392 was not interfered by p-gp expression and function in tumor cells (Figure 7B). Our data suggests that B392 is a strong microtubule binding agents but not a p-gp substrate.

## DISCUSSION

In this study, we reported a novel oral quinoline derivative, B392 as a potential drug candidate for anti-leukemia and multiple drug resistant cells. B392 dramatically inhibited leukemic cells survival; however, it had less cytotoxicity on normal cells *in vitro* and *in vivo*. The further evidences demonstrated that B392 is a depolymerizing MTA, which caused mitotic arrest. JNK played an essential mediator in B392-induced cells apoptosis through mitochondria disruption which ultimately leading to caspases activation. In addition, in sirolimus-resistant leukemic cells, B392 enhanced the cytotoxic effect of sirolimus; and the more importantly, it is not a substrate of p-gp.

Understanding the mechanisms of drug resistance to approved MTA, as well as their drawbacks in clinical therapy, is extremely important for the development of a promising and potent compound that targets microtubules. The main cause of MTA drug resistance in cancers is alteration of tubulin and overexpression of members of the ABC family, of which p-gp is the best known [8]. Several lines of evidence have revealed that vinca alkaloids and taxanes are pumped out by the membrane transporter p-gp, the product of multidrug resistance (*MDR)* gene and multidrug resistance-associated protein (MRP), thereby decreasing their intracellular concentrations and efficacy [26–28]. Therefore, after we demonstrated that B392 is indeed a microtubule destabilizing agent, as manifested by the result of our *in vitro* tubulin binding assay (Figure 3A), we also evaluated the susceptibility of B392 to p-gp in a p-gp-overexpressed NCI/ADR-RES cell line. We showed that NCI/ADR-RES cell was more sensitive to B392 than to vincristine or paclitaxel (Figure 7A) and the efflux of rhodamine did not decrease after co-incubation with a B392 (Figure 7B), implying that B392 might not be excluded by membrane transporter p-gp. Furthermore, most FDA-approved MTA for cancer treatment require parenteral administration; however, B392 was particularly designed for oral administration. The xenograft animal model demonstrated that antitumor efficacy after oral administration of B392 (Figure 2). Thus, B392 can be considered a potential and promising MTA for the treatment of leukemia.

We also performed a molecular docking analysis to identify the binding site of MPT0B392 on tubulin (Supplementary Figure 4). Tubulin has three main binding sites, namely colchicine, vincristine, and paclitaxel binding sites [29]. B392 was docked into each binding site using an in-house molecular docking tool, iGEMDOCK [30]. The docking results show that MPT0B392 occupied the colchicine binding site and exhibited the best docking score. In addition, the binding mode of B392 was similar to that of colchicine (Supplementary Figure 4). Both compounds contain the same functional group, 1,2,3-trimethoxybenzene, which is sandwiched by two hydrophobic residues L248 and L255 and forms stable van der Waals interactions with the residues. These observations suggest that B392 binds to the colchicine binding site and possesses a similar inhibition mechanism with colchicine.

Cell cycle progression is precisely regulated by cyclin and cyclin kinase complexes [31]. The activation of the cyclin B1/Cdk1 complex in the nucleus is responsible for entry into mitosis from the G2 phase. The observed upregulation of cyclin B1, decrease of phosphorylated Cdc2 at Tyr-15, and the phosphorylation of MPM2 demonstrated that B392 induced mitotic arrest and not G2 phase arrest (Figure 1D, 3C). In mitosis, if a chromosome attaches incorrectly to microtubule, the metaphase checkpoint (also called spindle assembly checkpoint, SAC) would be activated to elicit signal cascades to inhibit the APC, which causes cyclin B1 degradation and results in cells exiting mitosis [32]. In the present study, we found that after treatment with B392, the expression of Aurora A and Aurora B increased, and their downstream target H3S10 was phosphorylated, implying that B392 disrupted microtubule formation and might lead to SAC activation (Figure 3C). However, not every cancer cell with prolonged mitosis undergo apoptosis [33]. Anti-apoptotic mitochondrial proteins, Bcl-2, Mcl-1, and Bcl-xL, have been shown to be critical factors involved in the MTA-induced apoptosis pathway [34]. Knockdown of Mcl-1 can sensitize cells to spindle poison treatment [35] and overexpression of phospho-defective mutant Bcl-2 can block mitotic death [36]. In the present study, we found that B392 induced Bcl-2 phosphorylation and Mcl-1 decrease, which contributed to mitochondrial membrane potential loss, and caspase activation, indicating that B392 has actual cytotoxic abilities and does not merely increase cellular progression through mitosis.

Three MAPKs, ERK, JNK, and p38, have been found to be stress inducible in maintenance of cell functions. ERK was initially associated with cell proliferation and survival, whereas JNK and p38 were considered stress-inducible with effects on apoptosis. However, the mechanisms underlying MAPK regulation of apoptosis are complex and controversial, and might depend on cell type and drugs [23, 37]. Bcl-2, phosphorylated and inactivated by the JNK pathway, is normally activated in G2/M phase [38]. Therefore, we investigated to determine the specific MAPK that contributes to B392-induced apoptosis. Figure 5 shows that JNK inactivation can reverse B392-induced phosphorylation of Bcl2, decrease in Mcl-1, cell cycle distribution, and PARP and cleavage caspase 3. These imply that B392-induced JNK activation triggers the apoptotic pathway in leukemic cell lines.

Because mTOR signaling has proved to be crucial to cell proliferation and survival in hematological malignancies, mTOR inhibitors are being investigated in a myriad of preclinical and clinical trials [39]. However, the results of clinical studies suggest that some cancers have intrinsic resistance against these agents, while some have acquired resistance [40, 41]. According to our results, AML cell lines (HL60, MOLM-13, and MV4-11) show relative resistant to sirolimus when compared with ALL cell lines (MOLT-4 and CCRF-CEM) (Figure 6A). The synergism of B392 and sirolimus co-treatment was only observed in AML cell line (Figure 6B; Supplementary Table 1). It was reported that almost all AML samples have been observed to contain activated mTOR1, which is independent of PI3K/AKT [25]. Inhibition of mTOR1 by sirolimus would overactivate PI3K/AKT, thus producing AML cell resistance to sirolimus. It was also noted that downregulation of Mcl-1, a prosurvival member of the Bcl-2 family, can overcome resistance to sirolimus [42]. p-AKT and Mcl-1 expression decreased dramatically after B392 and sirolimus co-treatment (Figure 6C). Therefore, we hypothesized that the mechanism underlying this synergistic combination in AML cells might involve the inhibition of multiple pathways. Crazzolara. et al demonstrated that everolimus-vincristine co-treatment prolonged the survival of mice engrafted with ALL cells [43]. However, the combination of sirolimus and B392 had no effect on the ALL cell line, MOLT-4, in our *in vitro* study. We suggested that there might be more complicated mechanisms or factors influenced by the microenvironment that lead to this diversity.

Acute myeloid leukemia or acute lymphoblastic leukemia is a kind of high and poor risk leukemia, containing many subtypes based on distinct and complicated cytogenetic or molecular abnormalities, in contrast to chronic leukemia. Although conventional microtubule targeting drugs are regarded as an outdated chemotherapy, it cannot be denied that they can still have an impact on current anticancer therapy, as long as their deficiencies are improved. In the present study, we developed a novel oral quinoline derivative B392, that has an ability to inhibit tubulin polymerization and induce leukemic cell apoptosis though JNK activation. It is also less susceptible to p-gp activity, and enhances the cytotoxicity of sirolimus in sirolimus-resistant cells. Thus, B392 is considered a potential drug for the treatment of acute leukemia and multiple drug resistant cancers.

## MATERIALS AND METHODS

### Cell lines and biochemical regents

HL60 (acute promyelocytic leukemia), MOLT-4 (acute lymphoblastic leukemia) and CCRF-CEM (acute lymphoblastic leukemia), BEAS (normal human bronchial epithelium) and HUVEC (Human umbilical vein endothelial cell) were purchased from BCRC (Bioresource Collection and Research Centre, Taiwan); NCI/ADR-RES (National Cancer Institute/Adriamycin-Resistant, human ovarian carcinoma cell line) was obtained from the DTP Human Tumor Cell Line Screen; MV4-11(acute myeloid leukemia cell) and MOLM-13 (acute myeloid leukemia cell) were kindly gifted from National Health Research Institutes. HL60, MOLT-4, CCRF-CEM, BEAS, MV4-11, MOLM-13 and NCI/ADR-RES were maintained in RPMI-1640 medium (Invitrogen) supplemented with 10% FBS. HUVEC were grown in endothelial cell medium (ECM) (ScienCell Research Laboratory, Carlsbad, CA) supplemented with 20% FBS. All cell lines were incubated in 5% CO_2_ at 37°C. B392 was synthetized from Prof. Jing-Ping Liou (Taipei Medical University), and the purity is over 98%. Paclitaxel and vincristine were purchased from Sigma (St. Louis, MO). All of the above drugs were reconstituted in dimethysulfoxide (DMSO).

### Cell viability

The MTT (3- (4,5-cimethylthiazol-2-yl)-2,5-diphenyl tetrazolium bromide) assay was used to determine cell viability. The mitochondrial dehydrogenase of viable cells reduced 3-(4,5-dimethylthiazol-2-yl)-2,5-diphenyltetrazolium bromide, MTT (yellow) to insoluble formazan dyes (purple). Cells were seeded in 24-well (4 × 10^5^ cells/well) and then treated with B392 in 10% FBS-culture medium for 24 and 48 h. 100 μL mg/mL MTT solution (0.5 mg/mL in PBS) was added to the 24-well and incubated for 1 h at 37°C. The dyes were solubilized in the extraction buffer (0.1 M sodium acetate buffer, 100 mL/well). Finally, the absorbance (550 nm) was measured by spectrophotometer.

### Cell cycle analysis

Cells were seeded in six-well plates (8 × 10^5^/well) and treated with the drugs for the indicated time. Cells were collected, washed with PBS and fixed in 70% EtOH at −20°C for at least 30 min. Then cells were washed with PBS, treated with DNA extraction (0.2 M Na_2_HPO_4_-0.1M citritic buffer, pH 7.8) for 20 min and finally stained with propidium iodide (PI) solution (0.1% Triton X-100, RNAase A and PI) for 20 min. DNA contents were analyzed by flow cytometry using FACS Calibur (BD Biosciences).

### *In vitro* tubulin polymerization assay

Tubulin polymerization assay was conducted by using CytoDYNAMIX screen 03 kit (Cytoskeleton Inc., Denver, CO, USA). All steps were followed by manufacturer's protocol. Before preparing tubulin polymerization (TP) buffer, a 96 well-plate was pre-warmed in the Spectrophotometer at 37°C. TP buffer (including General Tubulin Buffer, Tubulin Glycerol Buffer and GTP), 85 μL/well and the test drugs (2 μL/well) were added to the pre-warmed 96-well plate, respectively. Here, DMSO was used for control, paclitaxel and vincristine for positive controls. Tubulin proteins were immediately added to the mixture (30 μL/well). The absorbance, 340 nm, were recorded every 1 min for 30 min at 37°C by ELISA reader (SpectraMAX Plus; Molecular Devices Inc., Sunnyvale, CA, USA).

### Immunofluorescence

Cells were seeded in round slide glass, pre-coated with poly-L-lysine, and treated with drugs for 24 h. Cells were fixed with 4% paraformaldehyde, permeabilized with 1% Triton X-100, blocked with 5% BSA and then incubated with α-tubulin primary antibody (Sigma-Aldrich, St. Louis, MO) overnight. In the next day, cells were washed with PBS and probed with appropriate secondary antibody for 1 h at RT (room temperature). Finally, cells were stained with mounting gel which contains DAPI then sealed with nail oil. Images were captured with the ZEISS, LSM 510 META confocal microscope.

### Tumor xenograft models

To evaluate *in vivo* antitumor activity of B392, MOLT-4 or HL60 cells (10^7^/mice) were injected into severe combined immunodeficient (SCID) mice subcutaneously. The following experiments were referenced by our published paper [18]. Mice were treated with indicated dosage of B392 and vincristine orally or intraperitoneally, when the average tumor size reached 100 mm^3^. The *in vivo* solvents of B392 and vincristine are 1% CMC (carboxymethyl cellulose) with 0.1% Tween 80 and 50% Cremophor EL with 50% DMSO, respectively. Body weights and tumor sizes were measured twice a week. The animal studies terminated when average size of the tumor was greater than 2,500 mm^3^. Tumor size was measured by caliper measurement (mm) and ellipsoid sphere formula (LW^2^/2, L: length; W: width). All procedures followed ethical standards and have been approved by National Taiwan University Animal Use and Management Committee (IACUC number: 20110303).

### Immunohistochemical staining

Tumor tissues were resected, immersed in formaldehyde, embedded in paraffin and sectioned. The detailed protocols were as described [44]. The sectioned slides were stained with the primary antibody cleavage caspase 3 or hematoxylin and eosin staining. HP-polymer conjugated secondary antibody (SuperPicture Polymer Detection kit) was used and then sectioned slides were stained with DAB Chomogen for 5 min. Mayer's Hematoxylin solution was used for counterstaining. Other sectioned slides in the same tissues were stained with hematoxylin and eosin (H&E), the color of nuclei was blue and of cytoplasm was pink.

### Western blot analysis

Cells were treated with drugs for indicated time and collected, washed with PBS. Pellets were lysed with RIPA (radioimmunoprecipitation assay) buffer containing protease and phosphatase inhibitors and under sonication. The supernatants were harvested. Protein concentrations were determined by BCA kit (Thermo Fisher scientific, Waltham, MA). The equal amounts of protein were loaded onto a sodium dodecyl sulfate-polyacrylamide (SDS-PAGE) gel then transferred to polyvinylidene difluorid (PVDF) membrane. The membranes were blocked for 1 h with 5% non-fat milk and probed with the primary antibodies of interest overnight at 4°C. The next day membranes were washed with TBST (Tris Buffered Saline with Tween^®^ 20) and subsequently incubated with appropriate horseradish peroxidase-conjugated secondary antibodies 1 h at RT. The antibody used in this study as follows : Cdc2 (pY15), Aurora B, caspase-8, caspase-9, p-mTOR, t-mTOR, p-AKT, t-AKT, p-P70S6K, P70S6K, p-4EBP, t-4EBP, p-JNK, t-JNK, p-P38, t-P38, p-ERK, t-ERK and internal controls, GAPDH and actin, were all purchased from Cell Signaling Technologies (Beverly, MA); Cyclin B, Cdc25C, Cdc2, PARP, Mcl-1, Bcl-2, pBcl-2, Bcl-xl and secondary antibodies were purchased from Santa Cruz (Santa Cruz, CA, USA); MPM2 (pSer/Th), H3 (pS10) were purchased from Upstate Biotechnology (Lake Placid, NY, USA); caspase 7 and caspase 3 were purchased from BD Bioscience (San Jos, CS, USA) and Imgenex (San Diego, CA, USA), respectively.

### Mitochondrial membrane potential loss

Rhodamine-123, a cell-permeant and green fluorescent dye with negative charged which can selectively bind to mitochondria membrane potential, was usually used to monitor mitochondrial membrane potential. Before cells were harvested, Rhodamine-123 was added then incubated for 30 min at 37°C. The results were analyzed by FACScan Flow Cytometer and CellQuest software (Bectman Dickinson)

### P-gp activity assay

Cells were treated with or without the indicated agents for 30 min and then co-treated with 10 μΜ Rhodamine-123 for 30 min at 37°C. Cells were trpsinized, washed with PBS and analyzed by flow cytometry (FACS Calibur, BD Biosciences).

### Statistical analysis

All experimental data were repeated at least three times and expressed as means (± SD). Statistical analysis was evaluated by student *t*-test, which was calculated to compare the mean of each group with that of the control group. *P*-values < 0.05 were represented statistical significance (*P < 0.05, ** *P* < 0.01, ***P < 0.001).

### Ethics approval and consent to participate

All *in vivo* procedures followed ethical standards and have been approved by National Taiwan University Animal Use and Management Committee.

### Consent for publication

All authors has read and approved the final manuscript.

## SUPPLEMENTARY MATERIALS FIGURES AND TABLE



## Abbreviations

MPT0B392: B392
MTA: microtubule-targeting agent
JNK: c-Jun N-terminal kinase
p-gp: p-glycoprotein
SAC: spindle assembly checkpoint
APC: anaphase-promoting complex
ABC: ATP-binding cassette
