# Supplementary material for: An oral quinoline derivative, MPT0B392, causes leukemic cells mitotic arrest and overcomes drug resistant cancer cells
Source: Oncotarget. 2017 Feb 6;8(17):27772–85. doi: 10.18632/oncotarget.15115 (PMC5438607; doi:10.18632/oncotarget.15115)
Supplement: Supplementary file 1 [file oncotarget-08-27772-s001.pdf]

An oral quinoline derivative, MPT0B392, causes leukemic cells mitotic arrest and overcomes drug resistant cancer cells

Supplementary Materials

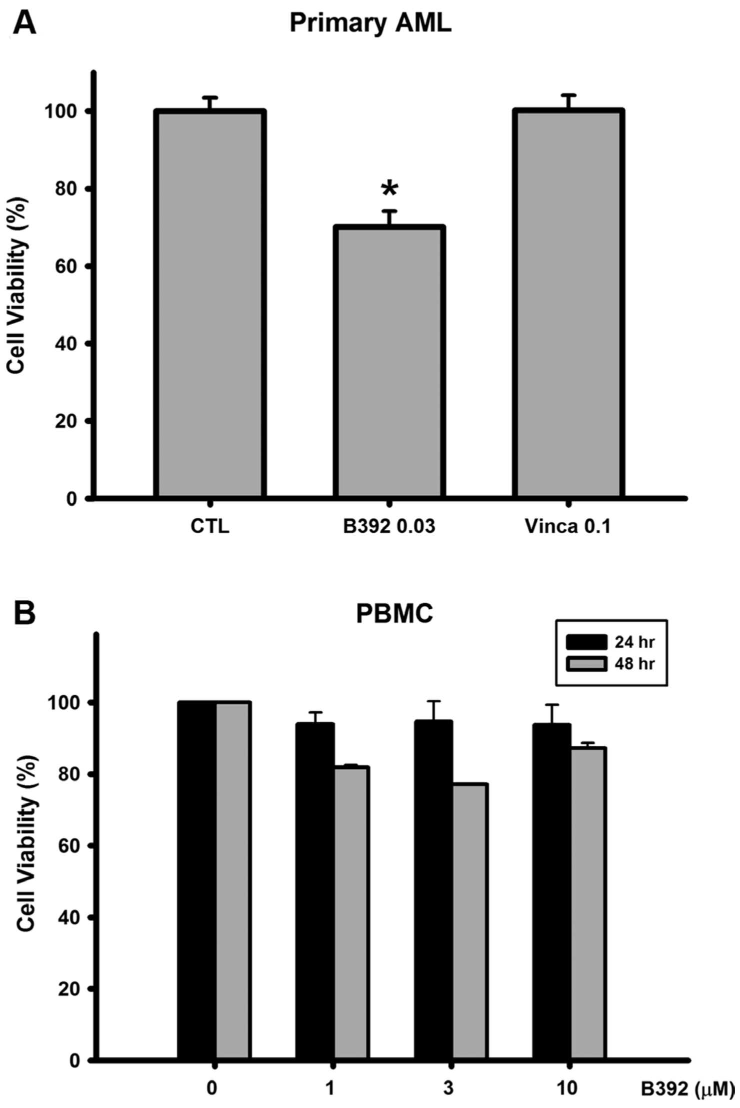

Supplemental Figure 1: The cell viability of B392 on primary AML cells and PBMC. (**A**) Primary AML cells (**B**) peripheral blood mononuclear cells (PBMC) were treated with indicated concentrations of B392, vincristine for 24 or 48 h. The cell viability was determined by MTT assay. (**P* < 0.05).

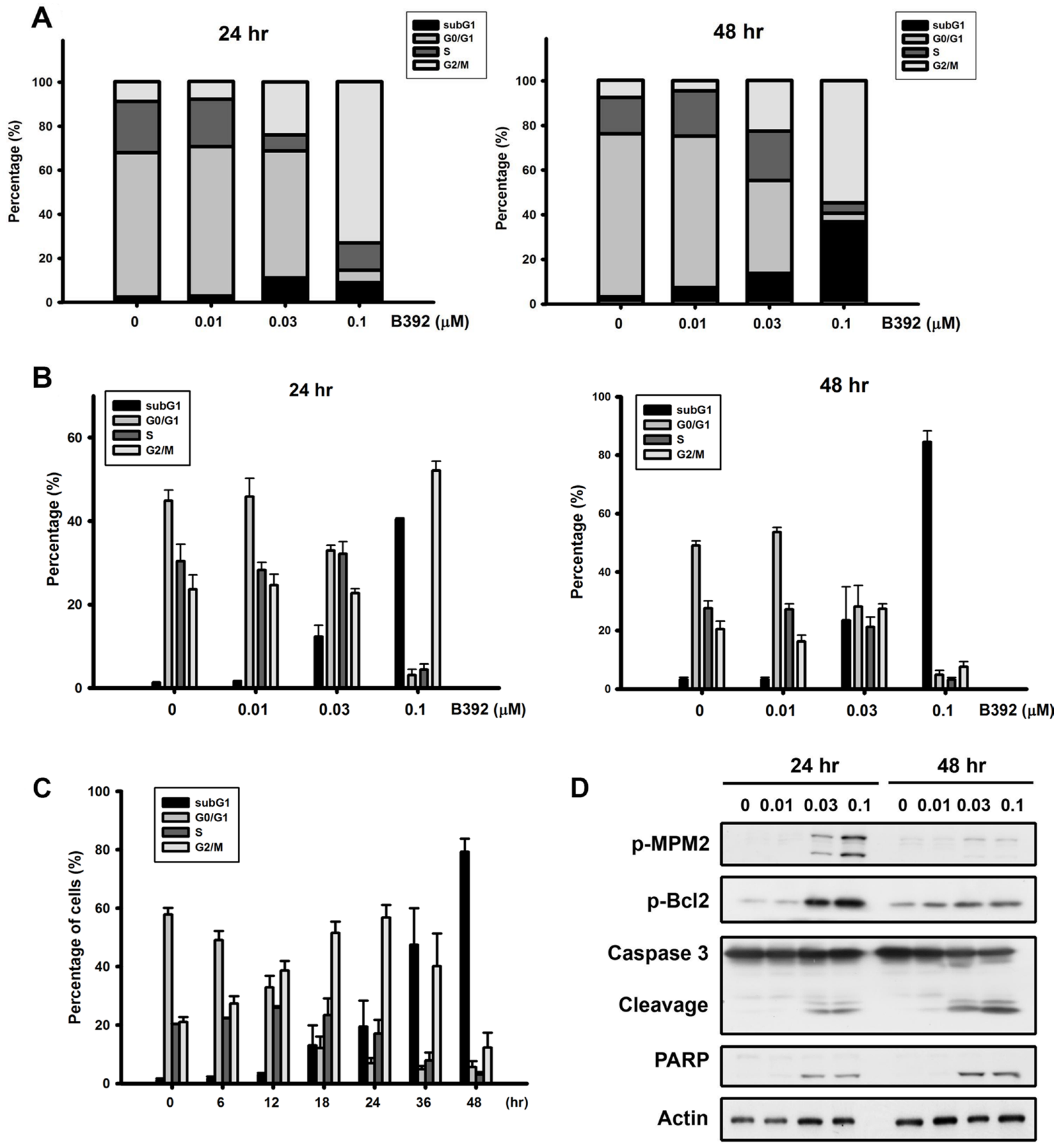

Supplemental Figure 2: The effect of B392 on MOLT-4 and CCRF-CEM cell lines. (**A**) MOLT-4 and (**B**) CCRF-CEM cells were incubated with B392 (0.01, 0.03, 0.1μM) for 24 h and 48 h, and then cells were harvested for (A & B) cell cycle analysis. (**C**) The statistic result of Figure 1D (left panel). (**D**) MOLT-4 cells were treated with indicated concentrations of B392 for 24 h and 48 h to detect the MPM2, p-Bcl-2, caspase 3 and PARP expression by western blot analysis.

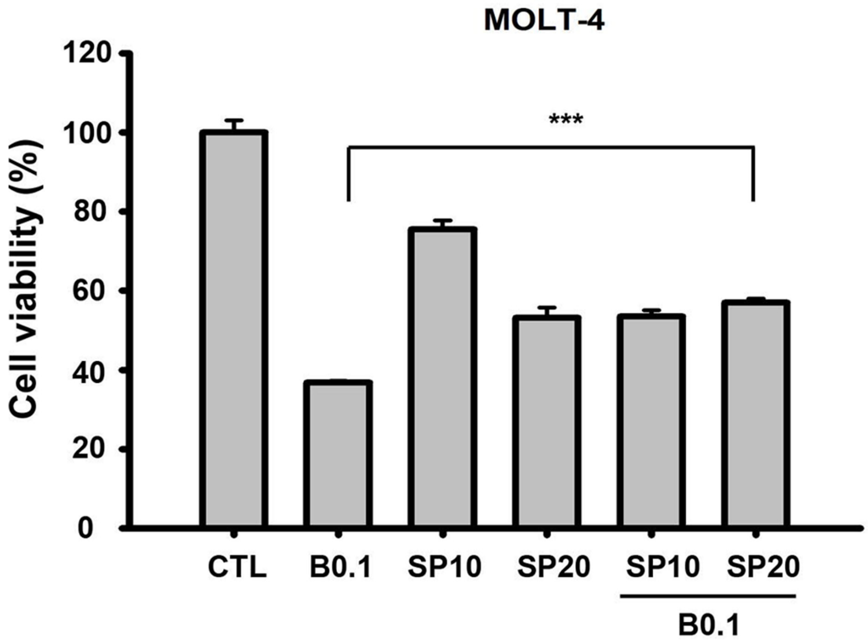

Supplemental Figure 3: B392 induced MOLT-4 apoptosis also though JNK activation. Cell viability of MOLT-4 cells after treatment with B392 at dose of 0.1 μM for 48 h in the absence or presence SP600125 (10, 20 μM). SP: SP600125, JNK inhibitor. ***$P < 0.001$.

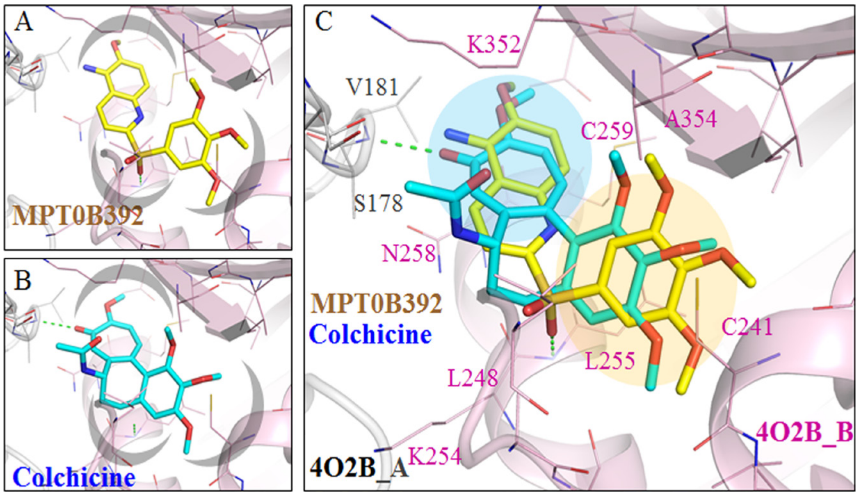

Supplemental Figure 4: Binding modes of B392. (**A**) Binding conformation of B392. The conformation is generated using a molecular docking tool, iGEMDOCK. The three-dimensional structure of tubulin used for docking is retrieved from the Protein Data Bank (PDB code 4O2B). (**B**) Binding conformation of colchicine, which is obtained from the complex structure (PDB code 4O2B). (**C**) Superimposed conformations of B392 and colchicine.

Supplementary Table 1: Combination B392 with sirolimus treatment displayed synergistic cell viability inhibition on HL60 cells

Dose (B392)	Dose (Sirolimus)	Effect	CI
**48 hr**			
0.03	1	0.0534	0.58096
0.03	5	0.0791	0.36194
0.03	10	0.293	0.06125
**72 hr**			
0.03	1	0.1605	0.65012
0.03	5	0.1536	0.67161
0.03	10	0.3076	0.38419

CI: Combination Index

The combination index (CI) was calculated based on the cell viability of B392 and sirolimus combination for 48 h on and 72 h HL60 cells. CI value less then 1 indicates synergy. The cell viability was determined by MTT assay.
